# Household size and its role in the association between multimorbidity and health and social care outcomes in older adults in Wales: retrospective cohort study

**DOI:** 10.1136/bmjmed-2024-001317

**Published:** 2025-11-30

**Authors:** Clare MacRae, Stewart W Mercer, Rhiannon K Owen, Rose Penfold, Stella Arakelyan, Chris Dibben, Jamie Pearce, Andrew Lawson, Nazir I Lone, Karin Modig, Bruce Guthrie

**Affiliations:** 1 Advanced Care Research Centre, University of Edinburgh Usher Institute, Edinburgh, UK; 2 University of Edinburgh Usher Institute, Edinburgh, UK; 3 Karolinska Institute, Stockholm, Sweden; 4 Swansea University Medical School, Swansea, UK; 5 University of Edinburgh School of GeoSciences, Edinburgh, UK; 6 Department of Public Health Sciences, Medical University of South Carolina, Charleston, South Carolina, USA

**Keywords:** Epidemiology, Public health, Health services, Healthcare Disparities

## Abstract

**Objective:**

To examine how the risk of unplanned admission to hospital and transitioning to live in a care home by number of long term conditions varies by household size.

**Design:**

Retrospective cohort study.

**Setting:**

Wales Census 2011 household data, linked to the Welsh Secure Anonymised Information Linkage (SAIL) Databank, 27 March 2011 to 26 March 2016.

**Participants:**

391 686 residents of Wales recorded in the Wales Census on 27 March 2011, aged ≥65 years, living in Welsh households of one to six residents, registered with a general practitioner contributing data to the SAIL Databank.

**Main outcome measures:**

Time to the first unplanned hospital admission and time to transition from living at home in the community to living in a care home, for individuals with 0-1, 2-3, or ≥4 long term conditions living alone or in households with two residents or three or more residents.

**Results:**

Of the 391 686 individuals included, 36.8% lived alone, 54.0% lived in households of two, and 9.2% lived in households with three or more people. The number of long term conditions was strongly associated with the risk of hospital admission and transition to a care home. In those living in two person households, participants with ≥4 long term conditions versus those with 0-1 long term conditions had a higher risk of unplanned hospital admissions (adjusted hazard ratio 2.51, 95% confidence interval (CI) 2.47 to 2.55; crude event rate 180.1 (95% CI 178.5 to 181.7) *v* 54.8 (53.9 to 55.7) per 1000 person years) and of transitioning to live in a care home (adjusted hazard ratio 2.57, 2.49 to 2.66; crude event rate 7.2 (6.9 to 7.5) *v* 1.40 (1.3 to 1.5) per 1000 person years). Household size was associated with an increased risk of both outcomes but more strongly with transition to a care home than unplanned hospital admission. The risk of unplanned hospital admissions was higher for people with 0-1 long term conditions who lived alone than for those who lived in a two person household (adjusted hazard ratio 1.19, 95% CI 1.17 to 1.22; crude event rate 74.9 (95% CI 73.4 to 76.4) *v* 54.8 (53.9 to 55.7) per 1000 person years) and for transitioning to live in a care home (adjusted hazard ratio 1.48, 1.42 to 1.54; crude event rate 5.4 (5.0 to 5.8) *v* 1.4 (1.3 to 1.5) per 1000 person years). The association between the number of long term conditions and both outcomes varied by household size. Individuals with 0-1 long term conditions and living alone showed a higher risk of transitioning to live in a care home than individuals with 2-3 long term conditions living in two person households.

**Conclusions:**

In this study, the number of long term conditions was strongly associated with the risk of hospital admission and transition to living in a care home, and this association was less pronounced among those living alone. The risk of transitioning to live in a care home was higher for people with 0-1 long term conditions who lived alone than for those with 2-3 long term conditions who lived in two person households. These findings emphasise the need for personalised strategies that reduce the risk of unplanned admissions to hospital and support independent living, and that consider both the degree of multimorbidity and household size.

**WHAT IS ALREADY KNOWN ON THIS TOPIC**

**WHAT THIS STUDY ADDS**

- The number of long term conditions was strongly linked to higher risks of hospital admission and transition to a care home, however household size modified this relationship with this effect being less pronounced for those living alone
- The risk of transitioning to live in a care home was higher for people with 0-1 long term conditions who lived alone than for those with 2-3 long term conditions who lived in two person households

**HOW THIS STUDY MIGHT AFFECT RESEARCH, PRACTICE, OR POLICY**

- Further research is needed to understand how household composition affects the health and care needs of older people
- Future studies should explore the roles of caregivers, types of care provided, and how care needs change over time
- In clinical practice and policy, these findings highlight the importance of supporting older adults who live alone or lack informal care

## Introduction

 Population ageing is a major challenge to health and care systems globally. With increasing age, individuals commonly accumulate multiple long term conditions, known as multimorbidity. People with multimorbidity can have complex health and care needs, including reduced functional status,^1^ clinical burdens such as the need to manage complex drug treatment regimens, multiple hospital appointments, and a higher likelihood of hospital admission^2 3^ and of transitioning to live in a care home.^4^ Unplanned hospital admission accounts for approximately a quarter of the entire budget for the NHS in England,^5 6^ and the rate of unplanned hospital admissions for people aged older than 65 years old is increasing, rising most steeply (by 3.8% annually) in those aged 90 years and over.^7^ Public expenditure on long term social care (most of which is spent on residential care) in high income countries is also rising and is expected to at least double by the year 2050 to about 3.0% of gross domestic product.^8^

Understanding which older people are at the highest risk of unplanned hospital admission and transitioning to live in a care home is important. As well as individual characteristics, the household context for older people might affect their likelihood of unscheduled hospital care or need for long term residential care.^9 10^ Living with a spouse is associated with improved physical and psychological health,^11^ and reinforcement of positive health behaviours.^12^ Conversely, the absence of support from household co-residents may result in greater social isolation and reduce resilience to both acute illness and chronic functional limitation.^13^ This finding is particularly important because it is becoming increasingly common for older adults live alone. For example, in the UK, the proportion of adults aged ≥65 years who lived alone increased from 46.9% in 2013 to 50.3% in 2023.^14^

Little research has been conducted on how the impact of the number of long term conditions that a person has might interact with the size of their household to modify their risk unplanned hospital admission and transition to care home.^15^ The combined effect of these characteristics may differ from the effect of each separately, and identifying those with the highest risk combinations of these features could be used to target scarce resources most effectively. Based on population data for Wales, we examined how the number of long term conditions affected the risk of unplanned hospital admission and transition to care homes for older adults and whether these associations varied for people living in households of different sizes.

## Methods

### Data sources

The overall design was a retrospective cohort study. The population comprised residents of Wales recorded in the decennial Wales Census on 27 March 2011 who were aged ≥65 years and living in Welsh households of one to six residents who were registered with a general practitioner contributing data to the Secure Anonymised Information Linkage (SAIL) Databank (80% of general practices and 83% of Welsh residents^16^). Residents of care homes at the study cross section date were excluded from the analyses. We linked census data to routinely collected anonymised administrative and electronic health data for eligible participants. Households where participants lived were defined by place of residence on that date and were identified through deterministic matching with 2011 Wales Census data provided by the Office for National Statistics.^17^ Online supplemental figure 1 shows the cohort selection flowchart and online supplemental table 1 provides an overview of the included datasets, variables, and linkage methods.

### Study outcomes

The two outcomes examined were time to the first unplanned hospital admission and time to transition from living at home in the community to living in a care home. We obtained data on unplanned hospital admissions between 27 March 2011 and 26 March 2016 from the Patient Episode Dataset for Wales (PEDW). Identifying unplanned admissions to hospital was done by including all unplanned inpatient admissions from several sources: general practice, emergency department, bed bureau, consultant clinic or domiciliary visit, NHS Direct, and emergency inpatient admission referrals made from any other referral source. Care home is an umbrella term for long term care settings in the UK, including residential care homes for older adults and care homes for older adults with nursing support. Both residential and nursing care homes provide 24 hour care and support for adults with a range of needs.^18^ For data on transitions to living in a care home, we used the Welsh Demographic Service Dataset (WDSD) residential address field to identify individuals whose address registered at their general practice recorded relocation from a community residential anonymised linkage field (RALF) to a care home RALF, determined by linkage to the Care Homes dataset. For both outcomes, we measured the number of days from the study index date to the first event over five years of follow-up.

### Household size

The Wales Census 2011 unique household identifier was used to determine the number of people who lived in the same household. Household size was categorised into households of one, two, or three or more residents. All individuals (including co-residents aged <65 years living in households of eligible adult participants) were accounted for in measuring household size. Online supplemental table 2 has a summary of the characteristics of co-residents younger than 65 years who were not included as study participants.

### Long term conditions and multimorbidity

We measured the presence of 46 active long term conditions on 27 March 2011 with methods described in our previous study that incorporated data from multiple sources (primary care and hospital inpatient condition coding, with prescription data and laboratory results) to maximise the completeness of ascertainment.^19^ The included long term conditions were chosen based on the results from a recent consensus recommendations for measuring multimorbidity,^20^ and were ascertained by using Read version 2 codes, laboratory and prescribing data available from the Welsh Longitudinal General Practice Dataset (WLGP), and international classification of diseases 10th revision (ICD-10) codes from the PEDW (online supplemental box 1 and tables 3 and 4). The number of long term conditions was categorised as up to one long term condition (ie, no multimorbidity^21^), and those with multimorbidity were further categorised as two to three long term conditions and four or more long term conditions. Online supplemental table 5 shows the prevalence of each included long term condition.

### Other variables

Age and sex were identified from the WDSD, with age incorporated as a categorical variable. Data for sex were taken from information in the WDSD rather than from patient reported gender. Socioeconomic position was determined by using the Welsh Index of Multiple Deprivation (WIMD) 2011, in its original form categorised as 10 equal groups (groups 1-10) and in our study made into five equal groups (groups 1-5) to avoid small numbers in the transition to care home analyses, allocated to each household in the study. The WIMD is a relative measure derived from small area data based on the postcode of the residence.^22^ Smoking, alcohol consumption, and body mass index were recorded from the general practice record (WLGP) with Read version 2 and EMIS codes, and classified into categorical variables for regression analyses (online supplemental tables 6-8). Ethnic group was determined from Census 2011 primary care and hospital inpatient data sources, categorised according to National Statistics Wales^23^ (Asian or Asian British, black, African, Caribbean or black British, mixed or multiple ethnic groups, white, and other ethnic group) (online supplemental box 2). Some individuals did not have recorded data for ethnic group, smoking, alcohol consumption, and body mass index. Given that this study used routinely collected data, individuals with, compared with those without, recorded data for these variables may be systematically different, reflecting lower primary care consultation and preventive healthcare use. The causes of missingness within these variables were likely heterogeneous, so missing indicator categories for missing values were included to allow for residual confounding.^24^

### Statistical analysis

First, we described the characteristics of the whole study cohort and then grouped by household size at the study index date (27 March 2011). We chose a priori to examine the interaction between the number of long term conditions and household size because we hypothesised that the effect of multiple long term conditions would be different in those living alone versus those living with other people who might provide support. For descriptive purposes, we present crude incidence rates, expressed as events per 1000 person years of follow-up, to summarise the overall frequency of each outcome in the population. We modelled associations between this interaction and the two outcomes (unplanned hospital admission and transition to a care home) with separate models for each. The models used multistate Cox proportional hazards to evaluate this risk. The reported results include the coefficients for the first transition, which represents either the move from home to the first unplanned hospital admission or the transition from home to a care home, depending on the model. Coefficients for the two additional transitions were not reported because they were not the focus of this study; these were home to death and unplanned hospital admission or care home to death (online supplemental figure 2).

The R package survival was used to analyse the Cox proportional hazards models. The data were prepared for multistate modelling with the msprep command in the mstate package. This process automatically censors the competing event (of death) at the time of death. The time to event was measured in days from the study index date to the first unplanned hospital admission or transition to live in a care home, and was used as the underlying timescale for the models, expressed as hazard ratios and adjusted hazard ratios. Two level random intercept models were used, where individuals were nested in households to account for the hierarchical structure of the data. Partitioning of the variance to report an intraclass correlation coefficient according to level within the multilevel model was not possible with the Cox proportional hazards model because this model does not have a residual sum of squares, and all intraclass correlation coefficients require a residual sum of squares.^25^ A visual assessment of Schoenfeld residuals confirmed that the proportional hazard assumption was met because no significant deviations from a zero slope were found.

Variable selection for each adjusted model was guided by directed acyclic graphs with a recommended method to visualise the interaction effect (online supplemental figures 3 and 4).^26^ Although ethnic group was identified as a confounder in the directed acyclic graph for the transition to care home models, this variable could not be incorporated into the models because of the small numbers of individuals with the outcome across the different ethnic groups. The coefficients from the adjusted analyses for the number of long term conditions and household size interaction were plotted on the natural log scale to account for the multiplicative nature of the hazard ratio.^27^ Because caring expectations and responsibilities are often based on gender,^28^ we performed subgroup analyses for men and women separately.

### Patient and public involvement

Patients and the public were involved in the design and conduct of the study and the results were disseminated to patient and public communities through the Advanced Care Research Centre, University of Edinburgh.

## Results

We included 391 686 community dwelling individuals, aged ≥65 years and living in Wales, in our analysis. More than half of the study participants (n=211 526, 54.0%) lived in households comprising two people and more than a third of participants (n=144 044, 36.8%) lived alone. Only 9.2% of participants (n=36 116) lived in households of three or more people (table 1). The characteristics of the participants, described according to the number of long term conditions, age, sex, and socioeconomic position, varied according to the size of their households. Participants who lived alone had the highest number of long term conditions (mean 3.7) compared with those living in two person households (mean 3.2) or in households with three or more people (mean 3.2). On average, individuals who lived alone were the oldest in the study population (median 77 years, interquartile range (IQR) 70-83), were mostly women (66.7%), and their socioeconomic position was nearly equally represented across the five WIMD groups. Participants living in two person households were younger than those who lived alone (median 72 years, IQR 68-77 years), were more commonly men (52.4%), and were more likely to live in areas with the highest socioeconomic position (26.0% in the highest and 13.4% in the lowest of the five groups of socioeconomic position). The age and sex of participants in households with three or more persons were similar to those in two person households, with a median age of 71 (IQR 67-77) years; 53.0% of this group were men. This group, however, showed more variation across the socioeconomic position groups than those living in two person households (21.0% lived in areas with the highest and 17.1% in areas with the lowest of the five groups of socioeconomic position).

**Table 1:** Characteristics of whole study population and according to size of household

Characteristics	Whole study population (n=391 686)	Household size		
		1 person (n=144 044, 36.8% of total cohort)	2 persons (n=211 526, 54.0% of total cohort)	≥3 persons (n=36 116, 9.2% of total cohort)
Age (years):				
Median (IQR)	73.0 (68-80)	77.0 (70-83)	72.0 (68-77)	71.0 (67-77)
65-69	121 830 (31.1)	30 996 (21.5)	75 783 (35.8)	15 051 (41.7)
70-74	94 947 (24.24)	29 004 (20.1)	57 092 (27.0)	8851 (24.5)
75-79	75 455 (19.26)	28 783 (20.0)	40 977 (19.4)	5695 (15.8)
80-84	53 689 (13.71)	26 407 (18.3)	23 891 (11.3)	3391 (9.4)
85-89	31 446 (8.03)	18 942 (13.2)	10 550 (5.0)	1954 (5.4)
≥90	14 319 (3.66)	9912 (6.9)	3233 (1.5)	1174 (3.3)
Sex:				
Men	177 811 (45.4)	47 914 (33.3)	110 772 (52.4)	19 125 (53.0)
Women	213 875 (54.6)	96 130 (66.7)	100 754 (47.6)	16 991 (47.0)
Socioeconomic position (group):				
1 (lowest)	62 082 (15.85)	27 460 (19.1)	28 443 (13.4)	6179 (17.1)
2	77 618 (19.82)	31 493 (21.9)	38 793 (18.3)	7332 (20.3)
3	82 298 (21.01)	29 876 (20.7)	44 588 (21.1)	7834 (21.7)
4	78 509 (20.04)	26 551 (18.4)	44 770 (21.2)	7188 (19.9)
5 (highest)	91 179 (23.28)	28 664 (19.9)	54 932 (26.0)	7583 (21.0)
No of long term conditions:				
Mean (SD)	3.4 (2.5)	3.7 (2.6)	3.2 (2.4)	3.2 (2.5)
0-1	98 430 (25.13)	30 438 (21.1)	58 138 (27.5)	9854 (27.3)
2-3	130 213 (33.24)	45 473 (31.6)	72 691 (34.4)	12 049 (33.4)
≥4	163 043 (41.63)	68 133 (47.3)	80 697 (38.1)	14 213 (39.4)

Data are number (%) unless indicated otherwise.

IQR, interquartile range; SD, standard deviation.

### Unplanned hospital admission

**Figure 1:**
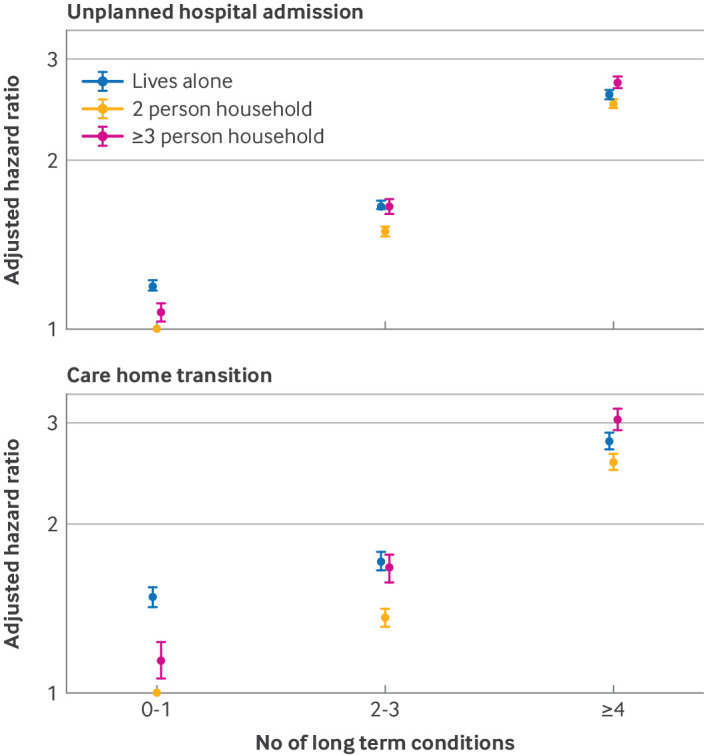
Adjusted hazard ratios (with 95% confidence intervals) for unplanned hospital admission and transition from home to live in a care home, by number of long term conditions, grouped by size of household

In the model examining unplanned hospital admissions, we included 1 434 813.8 person years of follow-up. Unplanned hospital admission was common, with a crude event rate of 123.7 first hospital admission events per 1000 person years across the whole study population. Individuals with up to one long term condition living in two person households were the lowest risk group and the reference for all reported hazard ratios (all of which were >1.00) (table 2). For all household sizes, people with more long term conditions were more likely to have an unplanned admission to hospital. This gradient was more substantial in the unadjusted models with a reduction when personal characteristics, health, and health behaviours were incorporated into the adjusted model. Among those living in two person households, participants with two to three long term conditions had a higher risk of unplanned hospital admission than participants with up to one long term condition (adjusted hazard ratio 1.49, 95% CI 1.46 to 1.52; crude event rates 88.4, 95% CI 87.3 to 89.5 *v* 54.8, 53.9 to 55.7 per 1000 person years); individuals with four or more long term conditions had a further increased risk (adjusted hazard ratio 2.51, 2.47 to 2.55; crude event rates 180.1, 178.5 to 181.7 *v* 54.8, 53.9 to 55.7 per 1000 person years). In the adjusted model, the difference in risk by number of long term conditions was less pronounced in those living alone and was larger in those living in two person households and in households with three or more people. For all groups of long term conditions (0-1, 2-3, and ≥4 long term conditions), living in a two person household was associated with the lowest rate and smallest adjusted hazard ratio for unplanned admission to hospital (figure 1). For those with the highest number of long term conditions, living in the largest households was associated with a higher risk of unplanned hospital admission than those who lived alone or in two person households.

**Table 2:** Unplanned hospital admissions according to number of long term conditions and size of household

No of long term conditions: household size interaction†	Event rate/1000 person years (95% CI)	Unadjusted hazard ratio (95% CI)	Adjusted hazard ratio (95% CI)*
Lives alone:			
0-1 long term conditions	74.9 (73.4 to 76.4)	1.47 (1.43 to 1.50)	1.19 (1.17 to 1.22)
2-3 long term conditions	118.9 (117.3 to 120.5)	2.16 (2.12 to 2.20)	1.66 (1.63 to 1.69)
≥4 long term conditions	233.4 (231.3 to 235.5)	3.88 (3.81 to 3.95)	2.61 (2.56 to 2.66)
2 person household:			
0-1 long term conditions	54.8 (53.9 to 55.7)	1.0 (reference)	1.0 (reference)
2-3 long term conditions	88.4 (87.3 to 89.5)	1.55 (1.52 to 1.58)	1.49 (1.46 to 1.52)
≥4 long term conditions	180.1 (178.5 to 181.7)	2.98 (2.93 to 3.03)	2.51 (2.47 to 2.55)
≥3 person household:			
0-1 long term conditions	58.2 (55.9 to 60.5)	1.07 (1.03 to 1.11)	1.07 (1.03 to 1.11)
2-3 long term conditions	95.7 (92.9 to 98.5)	1.72 (1.67 to 1.77)	1.65 (1.60 to 1.70)
≥4 long term conditions	195.4 (191.3 to 199.5)	3.36 (3.28 to 3.44)	2.74 (2.68 to 2.81)

P<0.001 for interaction of household size × number of long term conditions.

* Adjusted for age, sex, socioeconomic position, ethnic group, smoking, alcohol, and body mass index.

† Main effects and interaction fitted as one variable; reference for all is people with 0-1 long term conditions living in a two person household.

CI, confidence interval.

### Transitioning to live in a care home

In the model examining the rates of transitioning to live in a care home, we included 1 922 292.25 person years of follow-up. Transitioning to live in a care home was less frequent than an unplanned admission to hospital, with a crude event rate of 7.2 per 1000 person years in the whole study population.

The risk of transitioning to live in a care home increased stepwise according to the number of long term conditions (table 3). Living in two person households with two to three long term conditions was associated with a higher adjusted hazard ratio of transitioning to live in a care home than those in two person households with up to one long term condition (adjusted hazard ratio 1.36, 95% CI 1.31 to 1.41; crude event rates 3.00, 95% CI 2.8 to 3.2 *v* 1.40, 1.3 to 1.5 per 1000 person years); individuals with four or more long term conditions had a substantially higher hazard ratio (adjusted hazard ratio 2.57, 2.49 to 2.66; crude event rates 7.2, 6.9 to 7.5 *v* 1.40, 1.3 to 1.5 per 1000 person years). In the adjusted model, the difference in the magnitude of risk of transitioning to live in a care home between the number of long term conditions was smaller in those living alone and larger in those living in two person households and in households with three or more people. For all groups of long term conditions (0-1, 2-3, and ≥4 long term conditions), living in a two person household was associated with the lowest risk of transition to care home. The risk of transitioning to live in a care home was higher for people with up to one long term condition who lived alone, however, than for those who had two to three long term conditions who lived in a two person household (figure 1).

**Table 3:** Transitioning from home to live in a care home according to number of long term conditions and size of household

No of long term conditions: household size interaction†	Event rate/1000 person years (95% CI)	Unadjusted hazard ratio (95% CI)	Adjusted hazard ratio (95% CI)*
Lives alone:			
0-1 long term conditions	5.40 (5 to 5.8)	1.99 (1.91 to 2.08)	1.48 (1.42 to 1.54)
2-3 long term conditions	9.60 (9.2 to 10)	2.94 (2.83 to 3.04)	1.71 (1.65 to 1.78)
≥4 long term conditions	17.4 (16.9 to 17.9)	5.99 (5.80 to 6.18)	2.80 (2.71 to 2.90)
2 person household:			
0-1 long term conditions	1.40 (1.3 to 1.5)	1.0 (reference)	1.0 (reference)
2-3 long term conditions	3.00 (2.8 to 3.2)	1.67 (1.61 to 1.73)	1.36 (1.31 to 1.41)
≥4 long term conditions	7.2 (6.9 to 7.5)	4.01 (3.88 to 4.14)	2.57 (2.49 to 2.66)
≥3 person household:			
0-1 long term conditions	1.40 (1.1 to 1.7)	1.11 (1.03 to 1.19)	1.14 (1.06 to 1.23)
2-3 long term conditions	3.00 (2.6 to 3.4)	1.99 (1.88 to 2.11)	1.67 (1.57 to 1.76)
≥4 long term conditions	9.2 (8.5 to 9.9)	4.89 (4.68 to 5.10)	3.06 (2.93 to 3.20)

P<0.001 for interaction of household size × number of long term conditions.

* Adjusted for age, sex, and socioeconomic position (ethnic group could not be included because of small numbers).

† Main effects and interaction fitted as one variable; reference for all is people with 0-1 long term conditions living in a two person household.

### Sensitivity analyses

In subgroup analyses for men and women, we found similar overall patterns of association between the number of long term conditions and household size for both outcomes. When examining both unplanned admission to hospital and transition to living in a care home, the additional risk of living alone was similar for both men and women. Women living in households with three or more people, however, had a higher baseline hazard ratio for both outcomes than men (online supplemental tables 9 and 10).

## Discussion

### Principal findings

In this study, we found that the number of long term conditions was strongly associated with the move from home to the first unplanned hospital admission and with the transition from home to living in a care home. This association varied by household size, with the association being less pronounced in those living alone. Household size was associated with both outcomes, but more strongly with the transition to living in a care home. The risk of transitioning to live in a care home was higher for people with up to one long term condition who lived alone than for those with two to three long term conditions who lived in a two person household.

### Comparison with other studies

A recent systematic review found that multimorbidity correlated with hospital admission in older adults.^29^ Their analysis of 33 studies found that having two or more long term conditions, compared with up to one long term condition, was associated with a pooled odds ratio of 2.35 (95% CI 1.34 to 4.12) for the likelihood of admission to hospital. When multimorbidity was defined as having three or more long term conditions, the pooled odds ratio was 2.77 (1.83 to 4.20), although the overlapping groups and wide CIs in the study limit interpretability. Similarly, in a cohort study of older adults in Catalonia, Spain, the risk of transitioning to live in a care home also increased with the number of long term conditions (hazard ratio 1.1, 95% CI 1.1 to 1.1, for each additional long term condition).^30^

We found an association between household size and unplanned hospital admission, with individuals living alone having a small increased risk compared with those in households of two residents. This finding is consistent with results from a cohort study of older adults living in England,^31^ where individuals living alone had a rate ratio of 1.14 (95% CI 1.14 to 1.15) for unplanned hospital admission compared with those living in larger households. Our study found that the risk of transitioning to live in a care home was strongly associated with household size, with those living alone at a higher risk than those living in larger households. This finding supports other reports, such as a cohort study from Northern Ireland that found a reduced rate ratio for admission to a care home in individuals living in two person households versus those living alone (rate ratio 0.70, 95% CI 0.64 to 0.78).^4^

Individuals living alone are more likely to experience social isolation and do not receive social and practical support from household co-residents.^13^ Living alone might be associated with reduced resilience to chronic functional limitations, which are particularly relevant to long term care requirements. In our adjusted models, the difference in the risk of transitioning to living in a care home for individuals with varying numbers of long term conditions was smallest for those living alone and greatest for those living in the largest households. A possible explanation might be that the capacity of co-residents to provide care may become insufficient when care needs are higher. For example, a qualitative study explored the experiences of caregivers for older adults with multimorbidity in Canada, who reported feeling increasingly burdened, isolated, and unable to cope as the needs of the person they cared for increased.^32^ Triggers were the difficulties faced when dealing with fragmented health and care services, compounded by poor communication and coordination between service providers.

A cross sectional study examining carers of older adults in the US found that providing care and facilitating access to formal care was the most challenging for those with the highest number of conditions and the most complex care needs.^33^ Delivering care and support under these circumstances was perceived to cause increased strain and depression, which in turn resulted in lower self-efficacy and perceived ability to provide adequate care and support. Variation in household size showed a small association with unplanned hospital admission but a stronger association with transition to living in a care home. This finding suggests that living with other people offers some support needed to mitigate against hospital admission during acute illness but may provide greater resilience against long term illness and chronic functional limitation that eventually necessitates moving into a care home.

Our study found that in individuals with the highest number of long term conditions, the risk of both unplanned hospital admission and transition to living in a care home was highest in those who lived in the largest households than in those who lived alone or in two person households. This finding might be because, in larger households, particularly those with multiple generations living together, the potential for co-residents to provide care might be reduced. Adults in middle age often provide care simultaneously for older adults and children, termed sandwich carers, and might experience financial and emotional difficulties related to caregiving and role overload.^34^

### Strengths and limitations of this study

The strengths of our study include the use of population data with household characteristics deterministically defined by the national census, measurement of a large number of long term conditions, including all of those recommended for use in multimorbidity research,^20^ and identification of long term conditions from multiple data sources (primary care and hospital inpatient condition coding, and prescription and laboratory results) to maximise the accuracy of ascertainment.^19^ Also, including sensitivity analyses ensured robust evaluation of the results under different modelling assumptions and in men and women separately. Our original hypothesis was that caring roles might differ between men and women. In our study, we found that women and men had a similar increased risk of unplanned hospital admission and of transitioning to live in a care home if they lived alone than if they lived in larger households. For individuals living in the largest households, however, women with more long term conditions had a higher risk of both of these outcomes than men with the same number of long term conditions, which might reflect differences in care roles for men and women. For example, women with more long term conditions might provide care for others or receive less care from co-residents than men.

The study had several limitations. The long term conditions included in the study were derived from a curated list that does not reflect all human diseases. The included long term conditions, however, were developed through a formal process of consensus with stakeholders in the study of multimorbidity. We could not include HIV in the long term conditions because of data restrictions in the SAIL Databank. Household size was measured as a static variable at baseline, and individuals might change their living arrangements over time. Using the decennial census to study households (which restricts the study to defining households at one time point), however, meant that we could deterministically measure other individuals present in the same household at baseline. Also, we could not determine kinship relationships or quantify the provision or direction of care within households.

A range of sociodemographic and health risk factors were included in the study to adjust for confounding. Nonetheless, as with any observational study, other unmeasured residual confounding may be present. We created missing indicators to manage missingness in the variables ethnic group, smoking, alcohol consumption, and body mass index. This approach relies on assumptions that might not hold; for example, that no unmeasured confounding within missingness exists, that missing confounder values are conditionally independent of the variable, and that missing confounder values are conditionally independent of the potential outcomes.^35^ This approach was chosen because of the lack of potential ways to generate better imputed values with electronic health records. A simulation study examining the implications of varying approaches to imputation and missing data management based on electronic health records found that using the missing indicator, compared with multiple imputation methods, did not improve or worsen the overall performance of predictive models.^36^

In our study, we examined unplanned hospital admissions rather than analysing particular reasons for hospital admission. Comparing admissions to hospital by type (eg, admissions in the three main categories of ambulatory care sensitive conditions of vaccine preventable, acute, and chronic)^37^ could be a useful addition in categorising unplanned admissions to hospital to inform policy in a more specific way. Ambulatory care sensitive admissions account for only about 11% of all unplanned attendances,^38^ however, and therefore each category would represent a small proportion of hospital activity, limiting relevance to the use of emergency hospital care at a population level.

### Study implications

Our study of a large representative population cohort included deterministic allocation of individuals to households. This approach allowed us to identify household co-residents, but we could not determine kinship relationships or the provision of, or direction of, care within the households. Further development of large scale data infrastructures supporting a more granular and dynamic understanding of households is needed so that the implications of household composition for health and care use are better understood, and investigation of causal links can be examined. Our study used survival analysis to jointly incorporate information on whether and when the outcomes occurred.^39^ A useful extension of this work, however, would be to use count models to examine the number of unplanned hospital admissions, length of hospital admissions, and the risk of readmission to hospital, which could be driven by the characteristics of the individual and the household in which they live.

Further research is needed to better understand how caregivers respond to providing support for the health and care needs of older people with varying numbers of long term conditions and different definitions of multimorbidity. Exploration of the barriers and facilitators that caregivers face when these care needs increase acutely or more slowly over time is needed. A more granular examination of the effect of specific long term conditions within households might provide new findings to support particular patient groups. Understanding whether the observed effects vary by ethnic group would also be helpful. Our analysis was limited by small numbers, which prevented the inclusion of ethnic group in the adjusted models for transitioning to live in a care home. Replicating this research in more ethnically diverse datasets would allow for more robust adjustment by including ethnic group and for subgroup analyses to explore potential differences across ethnic groups.

Research is needed to develop complex interventions for older people living in the community with increasing health and care needs, to improve the management of chronic illnesses, and to support people living in their home for longer. Policies that prioritise support for older adults who live alone or lack access to informal caregivers, while also supporting individuals who provide informal care, should be considered. For example, support could include care delivered in the home, support programmes available in the local community, and technologies that support independent living. Researching and investing in innovative alternative care and housing models will help mitigate the declining ratio between the caregiver and care recipient that accompanies population ageing.

### Conclusions

In this study, the number of long term conditions was strongly associated with higher risks of admission to hospital and to the transition from home to living in a care home, but this association was less pronounced for those living alone. The risk of transitioning to live in a care home was higher for people with up to one long term condition who lived alone than for those with two to three long term conditions who lived in two person households. These findings emphasise the need for personalised strategies that reduce the risk of unplanned hospital admissions and support independent living, particularly among older adults with multimorbidity and those with no multimorbidity but living alone.

## Supplementary material

10.1136/bmjmed-2024-001317 online supplemental file 1

## Data Availability

Data may be obtained from a third party and are not publicly available.
