# Supplementary material for: Household size and its role in the association between multimorbidity and health and social care outcomes in older adults in Wales: retrospective cohort study
Source: BMJ Med. 2025 Nov 30;4(1):e001317. doi: 10.1136/bmjmed-2024-001317 (PMC12684141; doi:10.1136/bmjmed-2024-001317)
Supplement: Supplementary file 2 [file bmjmed-2024-001317.reviewer_comments.pdf]

PEER REVIEW HISTORY

BMJ Medicine publishes all reviews undertaken for accepted manuscripts. Reviewers are asked to complete a checklist review form and are provided with free text boxes to elaborate on their assessment. These free text comments are reproduced below.

ARTICLE DETAILS

**TITLE (PROVISIONAL)**	Household size and its role in the association between multimorbidity and health and social care outcomes: a retrospective cohort study of 391,686 older adults in Wales
**AUTHORS**	Macrae, Clare; Mercer, Stewart; Owen, Rhiannon K; Penfold, Rose; Arakelyan, Stella; Dibben, Chris; Pearce, Jamie; Lawson, Andrew; Lone, Nazir I; Modig, Karin; Guthrie, Bruce

VERSION 1 - REVIEW

**REVIEWER NAME**	Batty, Jonathan
**REVIEWER AFFILIATION**	University of Leeds, Leeds Institute for Data Analytics
**REVIEWER CONFLICT OF INTEREST**	None
**AI DISCLOSURE**	No
**DATE REVIEW RETURNED**	29-Jan-2025

**GENERAL COMMENTS**	It was my pleasure to review this manuscript, entitled "Impact of household size on the association between multimorbidity and health and social care outcomes: a multilevel population study of 391,686 older adults in Wales" (manuscript ID: bmjmed-2024-001317), which was submitted to BMJ Medicine as an original research article.<br><br>This manuscript describes the analysis of a national-level retrospective cohort of older individuals in Wales, using linked (primary and secondary care) medical record and 2011 census data (with follow-up from 2011 to 2016). Specifically, it evaluates the association between two exposures (household size and the presence of multiple long-term conditions; MLTC) on two outcomes (unplanned hospital admission and transition to residential care), adjusting for a number of confounders. It finds that household size and MLTC are associated with both outcomes: those that live alone, with a greater burden of multimorbidity were generally at greatest risk.<br><br>This manuscript is exceptionally well-written and tackles an important (and largely unaddressed) question using a sophisiticated (and appropriate) analytic strategy. In particular, the supplementary appendix contains a wealth of useful supporting information to justify the decisions made throughout this study and its key findings.<br><br>However, I believe that there are some important issues in terms of the philosophy, methodology and reporting of the paper that preclude publication in its current form. I will outline these below.<br><br>Major comments:<br>- The background and methodology of this paper suggest that this analysis is trying to answer a number of related causal questions:

	i.e., what is the effect of household size and MLTC burden on the risk of unplanned hospitalisation and transition to residential care, adjusting for confounders. As such, a causal approach should be taken, with a priori identification of confounders of the exposure-outcome relationship (e.g. age, sex, WIMD, ethnicity) and ideally, formalisation of the relationship between exposures, convariates and outcomes should be undertaken using a DAG. In the case of multi-state modelling, this should ideally done separately for each transition modelled. Given causal basis of this study (and the sophisticated analytic methods used) it was a surprise that ethnicity was identified as a probable confounder, but not included in the models based on its effect on model fit (specifically, AIC). Omission of a known confounder leads to confounding, regardless of the impact on the overall model fit. Given that this was conceptually expected to be a counfounder, it should have been included regardless.<br>- More information about the multistate modelling strategy is needed - ideally a diagram showing the modelled transitions for both models (unplanned hospitalisation and care home transition models) should be included in the supplementary appendix. What package was used to create these models? It is stated that the use of the multistate model accounts for the competing risk of death. This is only the case if those that die were appropriately censored in the first transition model (i.e. home -> first hospitalisation and home -> residential care). If this was the case, it should be stated.<br>- This study includes a number of key confounders that are frequently missing in routinely-collected data (e.g. BMI, smoking, alcohol intake). The proportion of individuals with missing data for each covariate should be explicitly reported in e.g. Table 1 or 2. Individuals missing one or more covariates would not have been included in the models - the final effective sample size for modelling should be explicitly reported. Even if a small number of individuals were missing data on each covariate, this could lead to the inadvertant exclusion of a large chunk of the study population overall. The methods for handling missing data should be stated and justified - i.e. if multiple imputation was not used, why not? If complete case analysis has been used (or inclusion of a "missing category", which I suspect) the limitations of this approach should be added to the discussion section.<br>- The presentation of the study results is an examople of the Table 2 Fallacy (see e.g. https://doi.org/10.1093/aje/kws412). The HRs for the confounders presented in Tables 2 and 3 have no logical interpretation in this modelling strategy and should be included.<br>- Multilevel modelling has been perforomed to account for the anticipated 'clustering' of risk within household units. Although the theoretical justification for this approach is sound (individuals living within a similar household should be expected to have more similar risks of hospitalisation/residential care transition compared with individuals from different households) the utility of this approach should also be empirically reported (i.e. through reporting the interclass correlation coefficient, or ICC). This would give an indication of the proportion of the variance in each outcome that attributable to the clusters.<br>- The ascertainment of long-term conditions performed in this study was performed using a subset of a list of conditions that emerged from a Delphi study conducted by the group previously (Ho et al, 2022). Appropriate justification of which conditions were included have been provided in Table S3, and these have been comprehensively ascertained using a mix of primary care (Read V2, prescription), secondary care (ICD-10, but not procedure codes).

	Although this seems appropriate, I think that comment should be made in the limitations section that the list of conditions reflects a curated list of conditions deemed to be 'important' that does not comprehensively reflect the entire human experience of disease (i.e. with many notable omissions). I also note some surprising inclusions, such as 'Chronic Lyme disease' - although this has not been ascertained in the current study. Some important conditions (e.g. HIV, chronic pain, brain tumours, chronic UTI, long-term MSK issues) could not be ascertained in the present study.<br>- A p-value for the interaction between the two exposures of interest in each model should be reported.<br>- Figure 1 shows that (somewhat contrary to what is described in the text) those in a 3+ person household have similar or greater risks of unplanned hospitalisation or care home transition than those living alone, particularly at greater multimorbidity burdens. This is not adequately explained: why would this be the case? Further exploration around this is needed.<br><br>Minor comments:<br>- The study type should be clarified as a retrospective cohort study.<br>- How was discrepant ethnicity data handled? I.e. where the ethnicity in the CENW is different from the WLGP and PEDW, or there are multiple different ethnicities recorded in a single data source? This should be added to Supplementary Box S2.<br>- Key to this analysis is how "unplanned" hospital admissions were ascertained. I note that this is included in Table S1, but I think it is of sufficient importance to be included in the main text.<br>- In the results section, the reporting of the average number of LTCs and age by household size should be done using medians (interquartile ranges) to reflect the likely skewed nature of these measurements.

**REVIEWER NAME**	Mah, Jamine
**REVIEWER AFFILIATION**	Dalhousie University
**REVIEWER CONFLICT OF INTEREST**	None
**AI DISCLOSURE**	No
**DATE REVIEW RETURNED**	16-Feb-2025

**GENERAL COMMENTS**	SUMMARY OF THE SUBMISSION<br>This manuscript is entitled Impact of household size on the association between multimorbidity and health and social care outcomes: a multilevel population study of 391,686 older adults in Wales. It “examined how the number of long-term conditions affects the risk of unplanned hospitalisation and transition to care homes for older adults and whether these associations varied for people living in households of different sizes“. It follows a cohort of residents aged 65 year and over in Wales identified with the 2011 Census and uses multiple sources of linked data. The primary analyses were a two level multistate Cox proportional hazards for outcomes of unplanned hospitalization and transition to care home separately. The results reported on 391,686 individuals, demonstrating the interaction of number of LTC conditions * household size was significantly associated with hospitalisations and care home transitions.<br><br>GENERAL COMMENTS<br>This is a well written, well organized cohort study that answers a

	clear research question through two objectives of interest to the global ageing phenomenon. The separate men and women analyses in the supplement are also commended. The results are interesting, clearly presented and explained. There are a few clarifications requested. The authors' responses to some additional thoughts about the discussion to stimulate reflection would be appreciated but not necessary to my overall recommendation that this work merits publication.<br><br>SPECIFIC POINTS<br>Background<br>o Please define "long-term social care" and "care home" for readers not from the UK as these may be used differently abroad.<br>o Define acronyms (e.g. UK or GP) on first use<br><br>Methods<br>o Please justify excluding ethnicity from the model solely based on AIC, as ethnicity can be a key confounder in the proposed research question. Bias reduction, not just model parsimony should guide co-variable selection. Perhaps the ethnic diversity of the sample was insufficient? May also comment in discussion.<br>o Please define "modest" for the readers as it is used several times. Do the authors refer to modest in regards to effect size? P value / confidence interval? Or as a synonym for small?<br>♣ E.g. "Household size was modestly associated with unplanned hospitalisation but more strongly associated with care home transition"<br>♣ E.g. This gradient was more substantial in the unadjusted models with modest attenuation when demographic, health, and health behaviours were incorporated into the adjusted model<br><br>Results<br>o Please check if accurate. In the paragraph transitioning to live in a care home: "Within each stratum of LTCs (0-1, 2-3, and ≥4 LTCs), living in a two-person household was associated with the lowest rate and smallest aHR of unplanned hospitalisation." Do the authors mean to write transition to care home?<br><br>Discussion<br>o Please clarify "However, in our adjusted models, associations between living alone and transition to a care home were largest in people with 0-1 conditions and minimal in people with ≥4 conditions." Table 3 suggests an increased HR when people live alone with >=4 conditions<br>o Please clarify "household size was only weakly associated with unplanned hospitalization but more strongly associated with the transition to living in a care home, suggesting that living with others provides less resilience in the face of acute illness than resilience in the face of chronic illness and chronic functional limitation." Doesn't a stronger relationship between household size and transition to living in a care home suggest less resilience as increasing household size is not able to cope hence the greater hazard of transiting to care home?<br><br>Reflection Points to consider in the Discussion:<br>o The authors explain the findings for living alone versus two household settings, but I would like to see an explanation of why the hazards for 3+ households seemed to have a similar risk to one person households. Or why three person households have greater risk than two people households.

	o The authors' findings that the age associations demonstrated the greatest risk of hazards for the two outcomes despite accounting for LTC conditions is interesting – can the authors' provide some explanations as to why this may be the case? From a frailty perspective, biological aging (e.g. long-term care conditions) often accounts for chronological age. With age greater than 90 having the strongest association with the study's outcomes, what else could be missing in the model or is this a matter of ageism?<br>o Any discussion points about why males and female hazards ratios did not differ significantly?<br>o Have the authors considered the most common LTCs or combinations of LTCs? This might be explored with a simple cross table with counts in a supplement. For example, living with asthma is very different than living with dementia. The combinations of LTCs 4+ may be interesting as well; for example, cancer may go with VTE (venous thromboembolism), peripheral neuropathy and anemia (all related to cancer) would be very different risk of hospitalization/care home than someone with coronary artery disease (CAD) plus heart failure plus lung disease (COPD) and alcoholism.<br><br>Discussion/Conclusion: Can the authors provide implications of this work beyond the need for more research. Given their conclusion (that tailored strategies are needed to reduce the risk of unplanned hospitalization and support independent living), can the authors' make suggestions using examples from the literature examples about tailored strategies based on their findings of household size and LTCs?<br><br>Supplementary Figure 1 - Please clarify the "Individuals living in households of size 1-6 residents" as this appears to be your inclusion rather than exclusion criteria in the main manuscript.<br><br>SUMMARY<br>• Significance to the field of aging research - good<br>• Originality – good<br>• Appropriateness of the approach – excellent<br>• Appropriateness of the statistical analyses - excellent<br>• Adherence to correct scientific nomenclature - good<br>• Appropriate literature citations - adequate<br>• Soundness of conclusions and interpretation – minor revisions<br>• Organization – excellent<br>• Adequacy of title and abstract - good<br>• Appropriateness of figures and tables - excellent

**REVIEWER NAME**	Perera, Rafael
**REVIEWER AFFILIATION**	University of Oxford, Primary Care Health Sciences
**REVIEWER CONFLICT OF INTEREST**	I have been working in the area of Multiple Long Term conditions for the last few years and I am PI on an NIHR Funded Programme of work.
**AI DISCLOSURE**	No
**DATE REVIEW RETURNED**	22-May-2025

**GENERAL COMMENTS**	bmjmed-2024-001317<br>Impact of household size on the association between multimorbidity and health and social care outcomes: a multilevel population study of 391,686 older adults in Wales<br><br>Stats Review:

	The manuscript provides results from an epidemiological study evaluating the impact of living arrangements (alone, with a partner, more than two people) and multiple long-term condition status (0-1 condition, 2-3 conditions, >4) on unplanned hospitalisation and care home transition. The authors find an association in both of these that is independent of the inclusion of some relevant potential confounders (age, gender, socioeconomic position, BMI, and alcohol use).<br><br>The paper is well written, and the analysis tools used are adequate. The data and general findings are presented well and in a balanced manner.<br><br>There is a significant issue with the way the analysis methods have been applied. The approach generally depends on the question being asked. In the present manuscript, the focus appears to be on the causal association, and although the authors are cautious in their arguments and conclusions, the way the models are constructed and presented is not consistent with this focus.<br><br>A simple way to present what the important exposure variables are and which are the potential confounders (expected) would be using DAGs. These will help clarify the important associations that they are interested in, as well as relationships with and between the relevant confounders (and potential data gaps, either completely missing or partially missing). One thing that is critical in this analysis is that there are significant imbalances in the relevant confounders when looking at the categories of living arrangements (age and gender in particular). Given this, further presentation and stratification (extension of their Figure 1) to explore these associations would be relevant.<br><br>An example of the choice of focus is that excluding ethnicity from the model, based on the optimisation of AIC, is not appropriate if the interest is in accounting for known confounders, such as ethnicity.<br><br>Regarding the final outputs, it is unclear why the models have not found a significant difference once stratified by gender. If the data were available for the type of hospitalisation (e.g. due to acute or chronic issue, which we know to be differentiated by gender), it might help clarify the reason for this (if the data are available).

VERSION 1 – AUTHOR RESPONSE

Comment	Response	Changes to text, tables, and figures, and position in the manuscript
Reviewer 1		

1.01 The background and methodology of this paper suggest that this analysis is trying to answer a number of related causal questions: i.e., what is the effect of household size and MLTC burden on the risk of unplanned hospitalisation and transition to residential care, adjusting for confounders. As such, a causal approach should be taken, with a priori identification of confounders of the exposure-outcome relationship (e.g. age, sex, WIMD, ethnicity) and ideally, formalisation of the relationship between exposures, covariates and outcomes should be undertaken using a DAG. In the case of multi-state modelling, this should ideally done separately for each transition modelled. Given causal basis of this study (and the sophisticated analytic methods used) it was a surprise that ethnicity was identified as a probable confounder, but not included in the models based on its effect on model fit (specifically, AIC). Omission of a known confounder leads to confounding, regardless of the impact on the overall model fit. Given that this was conceptually expected to be a confounder, it should have been included regardless.	See addition of directed acyclic graphs (DAGs) for the unplanned hospitalisation and transition to care home models.<br><br>See inserted text into the main manuscript and the inclusion of DAGs in the supplementary material.<br><br>Ethnicity was included in the unplanned hospitalisation models according to the DAG because the outcome was more frequent and there were no small numbers in the frequency of this outcome.<br><br>Please note that for the outcome of transitioning to live in a care home, the rate of the outcome was too small (<10) across categories of ethnicity and therefore SAIL advised that this variable could not be incorporated into the adjusted model.<br><br>Based in the new model outputs, all relevant coefficients have been modified throughout the main manuscript text and tables, and the supplementary material tables.	"Variable selection for **each adjusted model was guided by directed acyclic graphs (DAGs) using a recommended method to visualise the interaction effect (Supplementary Figures S3 & S4)."**<br><br>Main manuscript: page 7 lines 24-25<br><br>**"Although ethnicity was identified as a confounder in the DAG for the transition to care home models, it could not be incorporated into the models due to small numbers experiencing the outcome across the categories of ethnicity."**<br><br>Main manuscript: Page 7 lines 26-28<br><br>Supplementary Figure S3. Directed acyclic graph for

		unplanned hospitalisation.<br><br>Supplementary material: page 24<br><br><br>Supplementary Figure S4. Directed acyclic graph for transition to care home.<br><br>Supplementary material: page 25
1.02 More information about the multistate modelling strategy is needed - ideally a diagram showing the modelled transitions for both models (unplanned hospitalisation and care home transition models) should be included in the supplementary appendix.	Diagrams reporting the transitions for both models have been added.	"Coefficients for the two additional transitions were not reported because they were not the focus of this study; these were: i) home to death, and ii) unplanned hospitalisation or care home to death **(Supplementary Figure S2)**."<br><br>Main manuscript: page 7 line 12.<br><br><br>Supplementary Figure 2. Multistate model diagrams.<br><br>Supplementary material: page 23.
1.03 What package was used to create these models?	Details of the R package have been added.	**"The R package "Survival" was used to analyse the Cox PH models."**

		Main manuscript: page 7 lines 12-13.
1.04 It is stated that the use of the multistate model accounts for the competing risk of death. This is only the case if those that die were appropriately censored in the first transition model (i.e. home -> first hospitalisation and home -> residential care). If this was the case, it should be stated.	The multistate model incorporates death as a competing risk.	"**The data were prepared for multistate modelling using the msprep command in the mstate package. This process automatically censors the competing event (of death) at the time of the death.**"<br><br>Main manuscript: page 7 lines 13-15.
1.05 This study includes a number of key confounders that are frequently missing in routinely-collected data (e.g. BMI, smoking, alcohol intake). The proportion of individuals with missing data for each covariate should be explicitly reported in e.g. Table 1 or 2. Individuals missing one or more covariates would not have been included in the models - the final effective sample size for modelling should be explicitly reported. Even if a small number of individuals were missing data on each covariate, this could lead to the inadvertant exclusion of a large chunk of the study population overall. The methods for handling missing data should be stated and justified - i.e. if multiple imputation was not used, why not? If complete case analysis has been used (or inclusion of a "missing category", which I	We have described the process of managing the missing data for these variables and provided rationale.	"**In the ethnicity, smoking, alcohol consumption, and body mass index variables, some individuals did not have recorded data. Given that this study used routinely collected data, individuals without compared to those with recorded data for these variables may be systematically different, reflecting lower primary care consultation and preventive healthcare use. The causes of**

suspect) the limitations of this approach should be added to the discussion section.		**missingness within these variables were likely heterogeneous, so separate categories for the missing data were included to allow for residual confounding.[24]"**<br><br>Main manuscript: page 6 lines 26-32.
1.06 The presentation of the study results is an example of the Table 2 Fallacy (see e.g. https://doi.org/10.1093/aje/kws412). The HRs for the confounders presented in Tables 2 and 3 have no logical interpretation in this modelling strategy and should be included.	We have removed the coefficients for the confounder variables.	Removed text: "Incorporating the interaction between the number of long-term conditions and household size significantly improved the fit of models for both outcomes compared to models that included each variable separately. Individual information on sociodemographic characteristics and health behaviours was used to adjust for confounding. Incorporating ethnicity worsened model fit (increased model AIC); therefore, ethnicity was not included in the final models."

		Main manuscript.<br><br><br>Removed text: "Age was associated with the greatest increase in hazard, with more modest associations seen for sex, SEP, and health behaviours (Table 2)."<br><br><br>Removed text: "Advancing age (even more so than in the models examining unplanned hospitalisation) was strongly associated with transitioning to live in a care home, as was being a woman, but SEP and health behaviours were only modestly associated."
1.07 Multilevel modelling has been performed to account for the anticipated 'clustering' of risk within household units. Although the theoretical justification for this approach is sound (individuals living within a similar household should be expected to have more similar risks of hospitalisation/residential care transition compared with individuals from	During the development of this work, we sought advice regarding calculation of an interclass correlation coefficient (ICC) to partition the variance according to level for this type of model. We have been advised by Prof Terry Therneau, who developed the "Survival" package in R, who advised that it is not definable given that the Cox proportional hazards model has no residual sum of squares and all ICC definitions make use of the residual sum of squares.	"**Partitioning of the variance according to level within the multilevel model was not possible using the Cox proportional hazards model given that this model does not have a residual sum of squares,**

different households) the utility of this approach should also be empirically reported (i.e. through reporting the interclass correlation coefficient, or ICC). This would give an indication of the proportion of the variance in each outcome that attributable to the clusters.		**and all intraclass correlation coefficients require a residual sum of squares.[25]"** <br>Main manuscript: page 7 lines 19-22.
1.8 The ascertainment of long-term conditions performed in this study was performed using a subset of a list of conditions that emerged from a Delphi study conducted by the group previously (Ho et al, 2022). Appropriate justification of which conditions were included have been provided in Table S3, and these have been comprehensively ascertained using a mix of primary care (Read V2, prescription), secondary care (ICD-10, but not procedure codes). Although this seems appropriate, I think that comment should be made in the limitations section that the list of conditions reflects a curated list of conditions deemed to be 'important' that does not comprehensively reflect the entire human experience of disease (i.e. with many notable omissions). I also note some surprising inclusions, such as 'Chronic Lyme disease' - although this has not been ascertained in the current study. Some important conditions (e.g. HIV, chronic pain, brain tumours, chronic UTI, long-term MSK issues)	We have included this point as a limitation.	"**The LTCs included within the study were derived from a curated list that does not reflect the entire human experience of disease. However, the included LTCs were developed through a formal process of consensus with stakeholders in the study of multimorbidity. Within the measured LTCs, it was not possible to measure HIV due to data restrictions within the SAIL Databank."** <br>Main manuscript: page 12 lined 4-8.

could not be ascertained in the present study.		
1.09 A p-value for the interaction between the two exposures of interest in each model should be reported.		"**P-value for the interaction of Household size x Number of LTCs <0.001**"<br><br>Main manuscript: footnotes for Table 2 (page 14) and Table 3 (page 15).
1.10 Figure 1 shows that (somewhat contrary to what is described in the text) those in a 3+ person household have similar or greater risks of unplanned hospitalisation or care home transition than those living alone, particularly at greater multimorbidity burdens. This is not adequately explained: why would this be the case? Further exploration around this is needed.	We have reconfigured the results section to show that the text refers to the results as presented within Tables 2 & 3. Additional text has been included to describe the results as shown in Figure 2. We have added text to the results and discussion sections.	"**Among those with the highest number of LTCs, living in the largest households was associated with a higher risk of unplanned hospitalisation than those who lived alone or in two-person households.**"<br><br>Main manuscript: page 9 lines 10-12.<br><br>**"Our study found that amongst individuals with the highest number of LTCs, the risk of both unplanned hospitalisation and transition to living in a care home was highest in those who lived in the largest households compared to**

		**living alone or in a two-person household. This might be because in larger households, particularly those with multiple generations living together, there may be a dilution of the potential for co-residents to provide care. Reasons for this might relate to middle-aged adults providing care simultaneously for older adults and children, termed "sandwich carers", who experience a higher level of caregiving-related financial and emotional difficulties and role overload than carers.[35]"**<br><br>Main manuscript: page 11 lines 15-23.
1.11 The study type should be clarified as a retrospective cohort study.		"The overall design was a **retrospective** cohort study."<br><br>Main manuscript: page 5 line 3.
1.12 How was discrepant ethnicity data handled? I.e. where the ethnicity in the CENW is different from the	Further detail has been added to the "Ascertainment of ethnicity" section within the supplementary material.	"Ethnicity was primarily identified using Wales Census

WLGP and PEDW, or there are multiple different ethnicities recorded in a single data source? This should be added to Supplementary Box S2.		2011 – Welsh Records (CENW) using the categories made available by National Statistics Wales **given that these data are based on self-report.**[14] Within this dataset, ethnicity is coded as Asian or Asian British, Black, African, Caribbean or Black British, Mixed or multiple ethnic groups, White, Other ethnic group. Where not present within Wales Census data, ethnicity codes present in routinely collected data available in Welsh Longitudinal General Practice Dataset (WLGP) and Patient Episode Dataset for Wales (PEDW) were used.[15] **Where codes were discrepant, the CENW categorisation was prioritised, followed by WLGP and then PEDW.**"<br><br>Supplementary material: Supplementary Box S2.

<table>
<tr>
<td>1.13 Key to this analysis is how "unplanned" hospital admissions were ascertained. I note that this is included in Table S1, but I think it is of sufficient importance to be included in the main text.</td>
<td>The information regarding selection of unplanned hospitalisation has been inserted into the main manuscript Methods section.</td>
<td>“Ascertainment of unplanned hospitalisation was done by including unplanned inpatient admissions from the following sources: general practice, emergency department, bed bureau, consultant clinic or domiciliary visit, NHS Direct, and emergency inpatient admissions referrals made from any other referral source.”

Main manuscript: page 5 lines 18-21.</td>
</tr>
<tr>
<td>1.14 In the results section, the reporting of the average number of LTCs and age by household size should be done using medians (interquartile ranges) to reflect the likely skewed nature of these measurements.</td>
<td>We have changed the summary statistic for age (continuous) to median and interquartile range. However, the mean and standard deviation was a more useful summary of the number of LTCs (continuous) and therefore we kept this measure. Please see below the median and IQR for number of LTCs:

<table>
<tr><th></th><th>Whole population</th><th>lives alone</th><th>2-person household</th><th>3+ person household</th></tr>
<tr><td>LTCs (Median, IQR)</td><td>3 (1-5)</td><td>3.0 (2.0-5.0)</td><td>3.0 (1.0-5.0)</td><td>3.0 (1.0-5.0)</td></tr>
</table>
</td>
<td>Main manuscript Results section page 8 lines 17, 19-20, and 23.

Main manuscript Table 1: page 14.</td>
</tr>
</table>

Reviewer 2		
2.01 Please define "long-term social care" and "care home" for readers not from the UK as these may be used differently abroad.	Additional text added to the Methods section of the main manuscript.	"**"Care home" is an umbrella term for long-term care settings in the UK. Both residential and nursing care homes provide 24-hour care and support for adults with a range of needs.**[18]"<br><br>Main manuscript: page 5 lines 21-23.
2.02 Define acronyms (e.g. UK or GP) on first use	Acronyms have been expanded or written in full.	United Kingdom. Main manuscript: page 4 line 21.<br><br>General Practitioner. Main manuscript: page 5 line 6.
2.03 Please justify excluding ethnicity from the model solely based on AIC, as ethnicity can be a key confounder in the proposed research question. Bias reduction, not just model parsimony should guide co-variable selection. Perhaps the ethnic diversity of the sample was insufficient? May also comment in discussion.	See response 1.01.	
2.04 Please define "modest" for the readers as it is used several times. Do the authors refer to modest in regards to effect size? P value / confidence interval? Or as a synonym for small? E.g. "Household size was modestly associated with unplanned hospitalisation	We have amended our language to ensure that the description of effect sizes is clearer.	"Household size was **associated with both outcomes** but more strongly with care home transition"

but more strongly associated with care home transition"<br>E.g. This gradient was more substantial in the unadjusted models with modest attenuation when demographic, health, and health behaviours were incorporated into the adjusted model.		Main manuscript: page 3 line 16.<br><br>"This gradient was more substantial in the unadjusted models with attenuation when demographic, health, and health behaviours were incorporated into the adjusted model."<br><br>Main manuscript: page 9 lines 1-3.<br><br>"Household size was **associated with both outcomes** but more strongly with care home transition, and the risk of care home transition was similar for people with 0-1 LTCs who lived alone and those with 2-3 LTCs who lived in a two-person household."<br><br>Main manuscript: page 10 line 4.<br><br>"We found an association between household size and unplanned hospitalisation, where individuals

		living alone were at **small** increased risk compared to those in households of two residents."<br><br>Main manuscript: page 10 lines 16-18.
2.05 Please check if accurate. In the paragraph transitioning to live in a care home: "Within each stratum of LTCs (0-1, 2-3, and ≥4 LTCs), living in a two-person household was associated with the lowest rate and smallest aHR of unplanned hospitalisation." Do the authors mean to write transition to care home?	We have re-checked these results and think that what is written is correctly when describing the results for the unplanned hospitalisation model. Reviewing Table 2 column four (Adjusted HR) – when comparing the HRs within each of the three strata of LTCs, the lowest HR is for the individuals living in two-person households.	
2.06 Please clarify "However, in our adjusted models, associations between living alone and transition to a care home were largest in people with 0 -1 conditions and minimal in people with ≥4 conditions." Table 3 suggests an increased HR when people live alone with >=4 conditions	Please accept our apologies for this oversight. The text within the Discussion section of the main manuscript has now been amended.	"**In our adjusted models, the difference in the risk of transitioning to living in a care home across individuals with varying numbers of LTCs was largest for those living alone and minimal for those living in the largest households**."<br><br>Main manuscript: page 10 lines 29-31.
2.07 Please clarify "household size was only weakly associated with unplanned hospitalization but more strongly associated with the	We have re-written this section to improve readability.	"**Variation in household size showed a small**

transition to living in a care home, suggesting that living with others provides less resilience in the face of acute illness than resilience in the face of chronic illness and chronic functional limitation.” Doesn’t a stronger relationship between household size and transition to living in a care home suggest less resilience as increasing household size is not able to cope hence the greater hazard of transiting to care home?		**association with unplanned hospitalisation but a stronger association with transition to living in a care home. This suggests that living with other people offers some support needed to mitigate against hospital admission during acute illness, but may provide greater resilience against long-term illness and chronic functional limitation that eventually necessitate**

		**moving into a care home."**<br><br>Main manuscript: page 11 lines 9 -14.
2.08 The authors explain the findings for living alone versus two household settings, but I would like to see an explanation of why the hazards for 3+ households seemed to have a similar risk to one person households. Or why three person households have greater risk than two people households.	See response 1.10	
2.09 The authors' findings that the age associations demonstrated the greatest risk of hazards for the two outcomes despite accounting for LTC conditions is interesting – can the authors' provide some explanations as to why this may be the case? From a frailty perspective, biological aging (e.g. long-term care conditions) often accounts for chronological age. With age greater than 90 having the strongest association with the study's outcomes, what else could be missing in the model or is this a matter of ageism?	See response 1.06	
2.10 Any discussion points about why males and female hazards ratios did not differ significantly?	We have added additional description within the Discussion section of the main manuscript.	**"Our original hypothesis was that caring roles might differ across sexes. In our study, we found that women and men**

		**were at a similar increased risk of unplanned hospitalisation and of transitioning to live in a care home if they lived alone compared to living in larger households. However, for individuals living in the largest households, women with more LTCs were at a higher risk of both of these outcomes than men with the same number of LTCs, which might reflect differences in gender care roles. For example, women with more LTCs might provide care for others and/or receive less care from co-residents than men. ”**<br><br>Main manuscript: page 11 lines 30-34 and page 12 lines 1-3.
2.11 Have the authors considered the most common LTCs or combinations of LTCs? This might be explored with a simple cross table with counts in a supplement. For example, living with asthma is very different than living	This is an important point. However, this represents a large substantive piece of work including researching which conditions to examine followed by investigation using the method within the current manuscript applied to different combinations and is therefore outside the scope for one manuscript. We have added	“**A more granular examination of the effect of specific LTCs within households might present new findings to**

with dementia. The combinations of LTCs 4+ may be interesting as well; for example, cancer may go with VTE (venous thromboembolism), peripheral neuropathy and anaemia (all related to cancer) would be very different risk of hospitalization/care home than someone with coronary artery disease (CAD) plus heart failure plus lung disease (COPD) and alcoholism.	a reflection of this to the further research recommendation within the Discussion section.	**support particular patient groups."**<br><br>Main manuscript: page 12 line 34, page 13 lines 1-2.
2.12 Discussion/Conclusion: Can the authors provide implications of this work beyond the need for more research. Given their conclusion (that tailored strategies are needed to reduce the risk of unplanned hospitalization and support independent living), can the authors' make suggestions using examples from the literature examples about tailored strategies based on their findings of household size and LTCs?	We have added policy-specific recommendations to the Discussion section of the main manuscript.	"**Policies that prioritise support for older adults who live alone or lack access to informal caregivers and simultaneously support individuals who provide informal care should be considered, for example, care delivered in the home, support programs available within the local community, and technologies that support independent living."**<br><br>Main manuscript: page 13 lines 5-9.
2.13 Supplementary Figure 1 - Please clarify the "Individuals living in households of size 1-6 residents" as this appears to be your inclusion rather than	Thank you for highlighting this error. We have corrected the study cohort flow chart.	Supplementary material: Supplementary Figure S1.

exclusion criteria in the main manuscript.		
Reviewer 3		
3.01 There is a significant issue with the way the analysis methods have been applied. The approach generally depends on the question being asked. In the present manuscript, the focus appears to be on the causal association, and although the authors are cautious in their arguments and conclusions, the way the models are constructed and presented is not consistent with this focus.<br><br>A simple way to present what the important exposure variables are and which are the potential confounders (expected) would be using DAGs. These will help clarify the important associations that they are interested in, as well as relationships with and between the relevant confounders (and potential data gaps, either completely missing or partially missing).	See comment 1.01.	
3.02 One thing that is critical in this analysis is that there are significant imbalances in the relevant confounders when looking at the categories of living arrangements (age and gender in particular). Given this, further presentation and stratification (extension of their Figure 1) to explore these associations would be relevant.	Thank you for making this point. The covariates in Figure 1 are adjusted for age and sex and therefore further stratification would not be possible unless these variables were removed from the model. The sex stratified analyses presented in Supplementary Tables S9 and S10 provides assessment regarding the imbalance by sex. Please let us know if we have misunderstood this point.	
3.03 An example of the choice of focus is that	See response to comment 1.01.	

excluding ethnicity from the model, based on the optimisation of AIC, is not appropriate if the interest is in accounting for known confounders, such as ethnicity.		
3.04 Regarding the final outputs, it is unclear why the models have not found a significant difference once stratified by gender.	See response to comment 2.10.	
3.05 If the data were available for the type of hospitalisation (e.g. due to acute or chronic issue, which we know to be differentiated by gender), it might help clarify the reason for this (if the data are available).	Thank you for this important point. This work could consider using a comparison of admissions across the three main categories of the ambulatory care sensitive (ACS) conditions (vaccine preventable, acute, and chronic) and could be a useful extension that builds on an framework to categorise unplanned hospitalisations. However, ACS admissions account for only around 11% of all unplanned attendances and therefore each category would represent a small proportion of hospital activity. We have added a sentence to the limitations section.	"**Comparing hospitalisations by type, for example, admissions across the three main categories of ambulatory care-sensitive (ACS) conditions (vaccine-preventable, acute, and chronic),**[37] **could be a useful extension that builds on a framework to categorise unplanned**

		**hospitalisations to inform policy in a more specific way. However, ACS admissions account for only around 11% of all unplanned attendances,[38] therefore, each category would represent a small proportion of hospital activity, limiting relevance to the use of emergency hospital care at a population level.”** <br>Main manuscript: page 12 lines 17-24.

VERSION 2 – REVIEW

**REVIEWER NAME**	Batty, Jonathan
**REVIEWER AFFILIATION**	University of Leeds, Leeds Institute for Data Analytics
**REVIEWER CONFLICT OF INTEREST**	None
**AI DISCLOSURE**	No
**DATE REVIEW RETURNED**	28-Jul-2025

**GENERAL COMMENTS**	Many thanks for your responses to the queries I raised in the prior round of review, and taking the time to address these so comprehensively with further analysis and the changes made to the manuscript.<br><br>I have no further comments to make.

**REVIEWER NAME**	Perera, Rafael
**REVIEWER AFFILIATION**	University of Oxford, Primary Care Health Sciences
**REVIEWER CONFLICT OF INTEREST**	I work in the area of MLTC and have received NIHR (UK) funding for my team and me.
**AI DISCLOSURE**	No
**DATE REVIEW RETURNED**	03-Sep-2025

**GENERAL COMMENTS**	Stats Review:<br><br>I want to thank the authors for their detailed and comprehensive reply to all the editors and reviewers comments. I believe the manuscript is much improved and except for a couple of minor details, acceptable for publication.<br><br>The first issue I want to raise is an important discrepancy in the current understanding of the use of the missing indicator method as a way of reducing confounding. The reference the authors use indeed recommends the use of this approach when the missing group is likely heterogeneous.<br><br>(current reference)<br>24. Columbia Mailman School of Public Health. Missing Data and Multiple Imputation. Available at https://www.publichealth.columbia.edu/research/population-health-methods/missing-data-andmultiple-imputation [accessed July 2025]<br><br>However, general causal inference theory suggests this is not the case. This is highlighted in the paper by Blake in 2019 (DOI: 10.1002/bimj.201900041), where they set specific conditions under which the use of the missing indicator method would be valid. Given the nature of the question and the size of the data (and the lack of potential ways of generate better imputed values), I believe that the missing indicator method is probably the most efficient approach in this analysis but, as one of the reviewer's comment, its limitations and particularly in the context of its use in EHRs research deserved including in the Discussion (Limitations or potential weaknesses).<br><br>My second issue is that I believe the following text in the Discussion: "In our adjusted models, the difference in the risk of transitioning to living in a care home across individuals with varying numbers of LTCs was largest for those living alone and minimal for those living in the largest households" is either incorrect or at least unclear. The

	rest of the Discussion AND their Table 3 show a larger difference in HR within the >3-person household strata than in the Live alone strata. I think a simple edit should help clarify this point.

VERSION 2 – AUTHOR RESPONSE

**Response number**	**Reviewer/editor comment**	**Response to reviewer/editor**	**Manuscript location and amended text**
**Reviewer: Rafael Perera**			
Reviewer comment 1	The first issue I want to raise is an important discrepancy in the current understanding of the use of the missing indicator method as a way of reducing confounding. The reference the authors use indeed recommends the use of this approach when the missing group is likely heterogeneous.<br><br>(current reference)<br>24. Columbia Mailman School of Public Health. Missing Data and Multiple Imputation. Available at https://www.publichealth.columbia.edu/research/population-health-methods/missing-data-andmultiple-imputation [accessed July 2025]<br><br>However, general causal inference theory suggests this is not the case. This is highlighted in the paper by Blake in 2019 (DOI: 10.1002/bimj.201900041), where they set specific conditions under which the use of the missing indicator method would be valid. Given the nature of the question and the size of the	See additional text that describes the limitations of and reasons for taking this approach.	Discussion section Page 13, lines 22-32:<br><br>"**We created missing indicators to manage missingness in the ethnicity, smoking, alcohol consumption, and body mass index variables. This approach relies on assumptions that might not hold, for example, that there is no unmeasured confounding within missingness, that missing confounder values are conditionally independent of the exposure, and that missing confounder values are conditionally independent of the potential outcomes.[37] However, this approach was**

	data (and the lack of potential ways of generate better imputed values), I believe that the missing indicator method is probably the most efficient approach in this analysis but, as one of the reviewer's comment, its limitations and particularly in the context of its use in EHRs research deserved including in the Discussion (Limitations or potential weaknesses).		**chosen because of the lack of potential ways to generate better imputed values using electronic health records. Ehrig et al[38] studied the implications of taking varying approaches to imputation and the management of missing data what using electronic health records. They found that using the missing indicator compared to multiple imputation methods neither improved nor worsened the overall performance of their predictive models.[38]"**
Reviewer comment 2	My second issue is that I believe the following text in the Discussion:<br>"In our adjusted models, the difference in the risk of transitioning to living in a care home across individuals with varying numbers of LTCs was largest for those living alone and minimal for those living in the largest households" is either incorrect or at least unclear. The rest of the Discussion AND their Table 3 show a larger difference in HR within the >3-person household strata than in the Live alone strata. I think a	Please accept our apologies for this error. We have amended the text accordingly.	See Discussion section page 12, lines 5-6:<br><br>"In our adjusted models, the difference in the risk of transitioning to living in a care home across individuals with varying numbers of LTCs was **smallest** for those living alone and **greatest** for those living in the largest households."

	simple edit should help clarify this point.		
